# Effect of Short-Term Transcutaneous Vagus Nerve Stimulation (tVNS) on Brain Processing of Food Cues: An Electrophysiological Study

**DOI:** 10.3389/fnhum.2020.00206

**Published:** 2020-06-18

**Authors:** Martina A. Obst, Marcus Heldmann, Helena Alicart, Marc Tittgemeyer, Thomas F. Münte

**Affiliations:** ^1^Department of Neurology, University of Lübeck, Lübeck, Germany; ^2^Institute of Psychology II, University of Lübeck, Lübeck, Germany; ^3^Cognition and Brain Plasticity Group, University of Barcelona, Barcelona, Spain; ^4^Max-Planck-Institute for Metabolism Research, Cologne, Germany; ^5^Cluster of Excellence in Cellular Aging and Aging-Associated Diseases (CECAD), Cologne, Germany

**Keywords:** tVNS, food, ERP, P2 and N2, brain stimulation, human, healthy, Bayesian

## Abstract

**Background**: The vagus nerve plays an important role in the regulation of food intake. Modulating vagal activity *via* electrical stimulation (VNS) in patients and animal studies caused changes in food intake, energy metabolism, and body weight. However, the moderating impact of cognitive processes on VNS effects on eating behavior has not been investigated so far.

**Hypothesis**: We hypothesized that transcutaneous VNS (tVNS) affects food intake by altering cognitive functions relevant to the processing of food-related information.

**Methods**: Using a repeated-measurement design, we applied tVNS and a sham stimulation for 2 h on two different days in normal-weight subjects. We recorded standard scalp EEG while subjects watched food and object pictures presented in an oddball task. We analyzed the event-related potentials (ERPs) P1, P2, N2, and LPP and also examined the amount of consumed food and eating duration in a free-choice test meal.

**Results**: Significant differences between stimulations were observed for the P1, P2, and N2 amplitudes. However, we found no tVNS-dependent modulation of food intake nor a specific food-related stimulation effect on the ERPs. Further analyses revealed a negative relationship between P2 amplitude and food intake for the sham stimulation. Significant effects are additionally confirmed by Bayesian statistics.

**Conclusion**: Our study demonstrates tVNS’ impact on visual processing. Since the effects were similar between food and object stimuli, a general effect on visual perceptual processing can be assumed. More detailed investigations of these effects and their relationship with food intake and metabolism seem reasonable for future studies.

## Introduction

The obesity epidemic in developed countries is one of the most pressing health problems. Between 1975 and 2016, the worldwide prevalence of obesity almost tripled in adults, whereas in children and adolescents (5–19 years), it increased nearly five-fold [World Health Organization (WHO), 2020]. In contrast, current treatment approaches, such as behavioral interventions, pharmacological treatments, and bariatric surgery, show limited effectiveness, are costly, or are burdened with side effects (Butryn et al., 2011; Kakkar and Dahiya, 2015; Al-Najim et al., 2018). Effective treatment options for the management of body weight are relevant because increased body weight is associated with various diseases (Afshin et al., 2017). Therefore, additional treatment modalities are urgently needed.

Brain stimulation techniques might represent such an alternative. Possible procedures currently include invasive approaches such as deep brain stimulation (DBS) and vagus nerve stimulation (VNS) and non-invasive options like transcranial magnetic stimulation (TMS), transcranial DC stimulation (tDCS), and transcutaneous VNS (tVNS). The scarce evidence on these techniques in the treatment of obesity has been reviewed recently (McClelland et al., 2013; Gorgulho et al., 2014; Val-Laillet et al., 2015; Göbel et al., 2017; Johnson and Wilson, 2018). Remarkable effects on food intake and body weight were shown by DBS of the hypothalamus and nucleus accumbens, regions associated with energy homeostasis and reward processing, respectively (albeit in single cases or small case series). Yet, the TMS and tDCS affected food craving, but no evidence exists so far that these two techniques also influence food intake and/or body weight. VNS also showed promising effects in animal studies, but the results of human (replication) studies are inconsistent so far (Pelot and Grill, 2018).

However, the modulation of vagal nerve afferents using brain stimulation techniques appears to be promising for a variety of reasons. De Lartigue (2016) reviewed evidence that vagal afferent neurons provide a satiety signal to the brain but lose sensitivity to peripheral signals in obesity, leading to further ingestion of palatable food. Disrupting vagal afferent neurons can lead to hyperphagia and weight gain. Several studies which used invasive vagal nerve stimulation to modulate these processes reported effects on body weight, metabolism, and fat tissue activity in animal models (Roslin and Kurian, 2001; Sobocki et al., 2002, 2006; Bugajski et al., 2007; Gil et al., 2011; Banni et al., 2012; Han et al., 2015). In human patient studies targeting refractory epilepsy and treatment-resistant depression, weight loss has been reported (Burneo et al., 2002; Bodenlos et al., 2007; Pardo et al., 2007; Abubakr and Wambacq, 2008; Vijgen et al., 2013; Ghani et al., 2015).

Using functional MRI, it has been shown that VNS leads to widespread activation in several brainstem regions (ipsilateral NTS, bilateral spinal trigeminal nucleus, dorsal raphe, locus coeruleus, and contralateral parabrachial area, Chae et al., 2003). In particular, the parabrachial area projects to a variety of structures comprising the hypothalamus, the insular cortex, the limbic system, and frontal regions such as the lateral prefrontal cortex (Van Bockstaele et al., 1999). Those regions have been shown to be activated by VNS as well (Lomarev et al., 2002; Chae et al., 2003). Since the insular cortex and hypothalamus are known for their involvement in the regulation of ingestive behavior (Sainsbury and Zhang, 2012; De Silva et al., 2012), both structures are candidates for the mediation of the potential effect of VNS on body weight and food intake. Moreover, it is assumed that VNS induces brain satiety signals by mimicking anorexigenic hormonal signals transmitted by vagal afferents; this ultimately leads to decreased food consumption (animal: Val-Laillet et al., 2010; Banni et al., 2012; human: Bodenlos et al., 2007). In addition, it was shown that the satiety status modulates various cognitive functions which occur in the cognitive processing of food information (Carbine et al., 2018). However, it is still unclear whether VNS also affects these cognitive functions in addition to metabolic and neuronal effects.

A recent development is tVNS that operates *via* electrodes placed in the outer ear (cymba conchae) that stimulate the auricular branch of the vagus nerve which projects to the nucleus tractus solitarii (NTS, Ellrich, 2011). Just like VNS, tVNS showed a similar activation pattern in the aforementioned brainstem and (sub)cortical brain regions (Frangos et al., 2015), leading to the assumption that tVNS could carry the same potential for modulating body weight and food intake behavior.

As tVNS has been shown to impact cognitive functions (Jacobs et al., 2015; Colzato et al., 2018; Sellaro et al., 2018) including action selection (Jongkees et al., 2018) and cognitive control (Fischer et al., 2018), this technique appears to be suitable to study the relevance of alterations in the cognitive processing of food information and the impact on food intake.

Cognitive functions are an essential part of the regulation of food consumption. They constantly integrate metabolic signals indicating homeostatic requirements, motivational needs, and external information by neurocognitive processes (Ferrario et al., 2016). Using event-related potentials (ERPs), it has been shown that being in a hungry state already affects early attentional functions during visual processing of food-related information resulting in higher P1 and N1 amplitudes (Plihal et al., 2001; Schacht et al., 2016). Indicated by varying P2, P3, and late positive potential (LPP) amplitudes, the homeostatic status also impacts selective and higher-order attentional processes to food-related information (Carbine et al., 2018). Based on the known tendency of organisms to approach food, an increase in the food-associated Nogo-N2 amplitude can be seen as an indicator for inhibiting the immanent approach tendencies toward food items (Watson and Garvey, 2013; Kong et al., 2015; Carbine et al., 2017). In some studies, the cognitive control-related N2 component correlates negatively with the body mass index (BMI) and food consumption (Carbine et al., 2018). This suggests that a decline in inhibitory control may lead to increased food consumption and, accordingly, to increased body weight. Similarly, a positive correlation between BMI and P2 has been found which points to an increased allocation of attentional resources to food items in people with a high BMI (Carbine et al., 2018). However, only one out of ten studies reported a relationship between increased LPP and elevated BMI (Versace et al., 2016) indicating that cognitive processes are just one of many factors which influence ingestive behavior.

To gain a first insight into a potential effect of tVNS on food-related cognition and ingestive behavior, we conducted the current study in healthy participants receiving stimulation of the cymba conchae or sham stimulation at the outer upper ear for about 120 min. This was done in a blinded crossover design after having fasted from 6 pm the day before. During sham and verum stimulation, the varying processing of food and object pictures (control visual stimuli) was assessed using event-related potentials (ERPs).

Based on previous findings, we were expecting a differential effect of tVNS on ERPs to food vs. object pictures. We tested effects on different ERP components (N1, P1, N2, P2, P3, and LPP), as in the absence of previous similar tVNS studies we could not formulate a more specific hypothesis. Furthermore, food intake was assessed after the ERP session to test whether tVNS leads to a reduction in calorie intake. Statistical analyses were performed in terms of frequential and Bayesian statistics to get a reliable estimate of the effects.

## Materials and Methods

### Subjects

The procedures were approved by the local ethics committee prior to the study. Thirty-one healthy, right-handed subjects (15 women) were recruited *via* mailing lists from the university community and gave written informed consent to participate. All participants were compensated for their effort. Thirty participants exercised regularly between 1 and 7 h per week. Five participants indicated that they had been dieting at one point, but none of the participants was currently on a diet. Five participants were smokers (all <10 cigarettes per day). As visible in Table 1, the BMI for all subjects was within the normal range. It turned out that the women were distributed evenly across the cycle ruling this out as a nuisance factor in our results. Exclusion criteria were the presence of any psychological (e.g., depressive episode, eating disorders), neurological (e.g., seizure, migraine), and/or somatic (e.g., cardiac arrhythmia, diabetes) disorders; a BMI greater than 30 kg/m^2^; an age above 40 years; and any kind of irregular sleep cycle (sleeping disorder, shift-working) and/or diet style (low-carb, vegetarian/vegan, weight reduction diet). All subjects had a normal or corrected-to-normal vision.

**Table 1:** Descriptive sample statistics with mean (M) and standard deviation (SD) as well as statistical significance (p).

variables	mean (SD)	mean (SD)	test-statistic	*p*-value
Age (years)	23.0 (2.0)			
BMI (kg/m^2^)	22.5 (1.7)			
sportive Activity (week)	3.3 (1.7)			
	**Day 1**	**Day 2**		
Hungriness (VAS)	8.5 (3.6)	8.9 (3.2)	*z*^w^_(32)_ = −0.6	0.556
	**8 am Group**	**10 am Group**		
Hungriness (VAS)	8.8 (3.2)	8.6 (3.7)	*z*^mw^_(34,28)_ = 0.17	0.865
	**tVNS and 8 am**	**tVNS and 10 am**		
Eating Duration (h)	0.22 (0.1)	0.19 (0.1)	*z*^mw^_(16,13)_ = 0.15	0.124
	**sham and 8 am**	**sham and 10 am**		
Eating Duration (h)	0.21 (0.1)	0.21 (0.1)	*z*^mw^_(16,13)_ = 0.15	0.878

**z*^w^ = Wilcoxon sign rank test for paired samples (two-sided). *z*^mw^ = Mann-Whitney-Wilcoxon test for unpaired samples (two-sided). *n* = 31; females = 15*.

### Procedure

Participants took part in two sessions (experimental stimulation, control stimulation) which were spaced 1 week apart. The order of the sessions was counterbalanced across participants. The participants were required to abstain from eating beginning at 18:00 h on the evening before the experimental sessions. They appeared either at 8:00 h or at 10:00 h in the laboratory. Participants indicated their subjective hungriness on a visual analog scale of 15 cm length. As visible in Table 1, there was no difference in the stated hungriness rating between each session (within-subject) as well as in terms of the measurements starting time (8 am vs. 10 am; between-subject). Also, the starting time point did not influence the eating duration in the final test meal.

### Transcutaneous Vagal Nerve Stimulation

**Figure 1:**
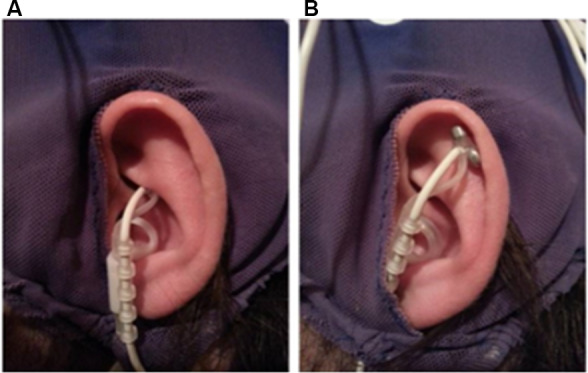
Positions of the ear-electrode (NEMOS^®^, Cerbomed Erlangen-Germany). **(A)** Transcutaneous stimulation of the auricular branch of the vagus nerve (tVNS), **(B)** stimulation of the auricular scaphoid fossa (sham). Stimulation parameters: 25 Hz, biphasic, 30 s ON and 30 s OFF interval, current intensity of 0.6 mA so that a tingly was perceptible.

Before the application of the EEG cap and ECG electrodes, a titanium/titanium–iridium ear electrode (Nemos^®^, Cerbomed, Germany) was applied to the cymba conchae of the left ear to stimulate the auricular branch of the vagus nerve transcutaneously (experimental condition, Figure 1A). As a control stimulation, the outer upper ear was targeted (Figure 1B). This region does not contain vagal afferents (Berthoud and Neuhuber, 2000). In both conditions, stimulation was performed with 0.6 mA and a frequency of 25 Hz and a biphasic impulse interval (30 s stimulation, 30 s pause). This stimulation leads to a tingling sensation at the ear. To enhance the contact between skin and electrode, a contact spray was used (TIGA-TRONIC, Tiga-med, Ronneburg, Germany). The total duration of the stimulation was 1.9 h (SD 0.2) in both conditions.

### Visual Stimuli and Procedure

**Figure 2:**
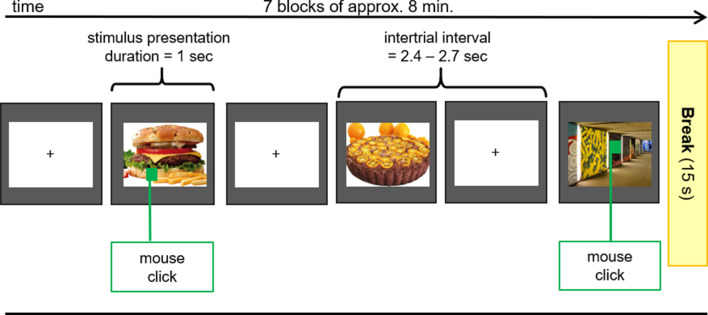
EEG Oddball-Paradigm. During the EEG-Measurements pictures of food with high and low caloric content as well as unexceptional objects (control) were presented to the subjects. If the presented picture contained a green square, participants had to press the right mouse button with the index finger of the right hand as fast as possible.

After the application of all electrodes, participants were seated in a comfortable chair in front of a video monitor (viewing distance 90 cm). They viewed a sequence of 140 pictures (duration 1,000 ms, intertrial interval 2,400–2,700 ms), subtending 20 (height) by 20 (width) degrees of visual angle. The picture set comprised 70 different food (sweet and savory, high and low caloric) and 70 different object pictures (e.g., household objects, items from nature) that were repeated seven times in a random order in seven blocks of approximately 8 min in duration (Figure 2). Between blocks, short breaks were given to allow the participants to stretch and move. Each block contained 10 pictures (both object and food items) that contained a small green square (1 × 1 cm) that could appear anywhere within the picture. The participant’s task was to screen each picture for the presence of the green square and to press a button whenever a square was detected. This ensured attentive processing of all pictures.

### Food Intake/Standardized Meal

After removing EEG and ear electrodes, the participants received a standardized breakfast containing a variety of foods typical for a continental breakfast (no meat; Supplementary Table S1). They were instructed to eat as much and as long as they wanted until they were satiated. To calculate the consumed amount of food, we calculated the difference between the food’s weight before and after breakfast. Also, the duration of food consumption was documented. For the analysis, the energy density of the consumed food was calculated using the *fddb database*^1^.

### EEG Recording and Analysis

The electroencephalogram was recorded from 29 scalp channels (FP1, FP2, F3, F4, C3, C4, P3, P4, O1, O2, F7, F8, T7, T8, P7, P8, FZ, CZ, PZ, FC1, FC2, CP1, CP2, PO3, PO4, FC5, FC6, CP5, CP6) referenced to the nose tip (bandpass of 0.01–50 Hz, 500 samples/s). Ocular fixation was verified by recordings of the horizontal EOG. Trials which were contaminated by eye blinks were detected by vertical electrooculogram.

EEG data were processed using EEGlab (Delorme and Makeig, 2004) and ERPlab (Lopez-Calderon and Luck, 2014) implemented in MatLab R2017a (MathWorks Inc.). First, frequencies below 0.1 Hz and above 48 Hz were filtered out. Next, the data were divided into 3,000-ms epochs starting 1,000 ms before the onset of the picture presentation. Independent component analysis (ICA) was used to remove ocular and muscle artifacts from the data. Briefly summarized, ICA separates the signal into different statistically independent sources. Sources identified as artifactual were removed (Bell and Sejnowski, 1995; Makeig et al., 1996). Additionally, artifact-contaminated epochs were rejected based on the identification of peak-to-peak amplitudes exceeding 130 μV and visual inspection. Less than 20% of the epochs were rejected per participant. The remaining epochs were used to calculate an average ERP per subject and condition.

For statistical testing, mean amplitudes with a baseline of −100 to 0 ms were calculated using component-specific time windows for early (P1 100–140 ms, N1 140–180 ms), middle (P2 210–260 ms, N2 260–360 ms), and late components (LPP 400–600 ms). P1 and P2 mean amplitudes were calculated for the occipital–parietal scalp regions (P3, P4, PO3, PO4, O1, O2), N1 and N2 mean amplitudes for midline electrode positions (FZ, CZ, PZ), and the mean amplitudes LPP at the electrode sites C3, C4, P3, P4, O1, and O2.

Each component was analyzed separately. For P1, P2, and LPP, a 3 (gradient: parietal, occipital–parietal, occipital) × 2 (hemisphere: left vs. right) × 2 (stimulation: tVNS vs. sham) × 2 (picture: food vs. object) repeated-measures ANOVA and for the N1 and N2 components a 3 (electrode: FZ, CZ, PZ) × 2 (stimulation: tVNS vs. Sham) × 2 (picture: food vs. object) repeated-measures ANOVA were calculated using IBM SPSS (Version 22). All tests were conducted two-sided at the 5% significance level and adjusted for multiple comparisons using Bonferroni correction. Effect sizes were reported as partial *η*^2^ for ANOVAs and Hedges g for *post hoc* paired-sample t-tests. To assess stimulation effects on food consumption, two-sided paired-sample t-tests on a 0.05 alpha level were conducted in SPSS and effects sizes are reported (Hedges g).

Following Colzato et al. (2018) and Warren et al. (2019), we additionally used Bayesian statistics (Kennedy, 2015; Wagenmakers et al., 2016, 2018) to further evaluate the significance of effects. In contrast to frequential statistics, one benefit of Bayesian statistics is the calculation of the Bayes factor (BF) representing the strength of evidence for the null (H0) and the alternative hypothesis (H1) given by the empirical data. Bayesian statistics is also known to be more conservative when testing for the alternative hypothesis (Gelman et al., 2012; Wagenmakers et al., 2018) and can accordingly be seen as a lower limit of the effect’s strength, providing further evidence for the validity of the reported findings.

Bayesian paired-sample *t*-tests for *post hoc* analysis of the ANOVA effects were calculated using the JASP software package (JASP v0.8.6.0).^2^ For these statistics, the prior probability was defined by a Cauchy distribution (default setting) and the BF (H1|H0) is reported. The BF(H1|H0) is the ratio that quantifies the likelihood of H1 over H0; i.e., a BF_(10)_ of three means that H1 is three times more likely (based on the empirical data) than H0. According to Kass and Raftery (1995), a BF between one and three indicates anecdotal, between three and ten moderate, between 10 and 30 strong, and between 30 and 100 very strong evidence for H1. Potential interrelations between electrophysiological effects and food consumption were tested using Pearson correlation and Bayesian correlation pairs, respectively.

## Results

### Electrophysiological Results

Descriptive statistics (mean, standard error, and 95% confidence interval values) are reported in Table 2.

**Table 2:** Descriptive statistic for component analysis.

	picture				stimulation			
		**M(SE)**	*p*	**CI_Difference_**		**M(SE)**	*p*	**CI_Difference_**
P1	food	6.61 (0.61)	0.055^t^	−0.01 0.86	*tVNS*	6.18 (0.61)	0.028*	−0.78 −0.05
	object	6.20 (0.60)			*Sham*	6.63 (0.60)		
N1	food	0.67 (0.43)	0.587	−0.20 0.34	*tVNS*	0.50 (0.45)	0.119	−0.60 0.07
	object	0.60 (0.41)			*Sham*	0.77 (0.40)		
P2	food	15.94 (1.13)	<0.001^***^	1.36 2.38	*tVNS*	14.05 (1,12)	0.018^*^	−1.85 −0.18
	object	13.63 (1.02)			*Sham*	15.07 (1.05)
N2	food	0.88 (0.56)	<0.001^***^	2.75 3.39	*tVNS*	−0.91 (0.58)	0.012^*^	−0.89 −0.12
	object	−2.19 (0.55)			*Sham*	−0.40 (0.53)
LPP	food	7.26 (0.57)	<0.001^***^	0.62 1.23	*tVNS*	6.48 (0.59)	0.059^t^	−1.30 0.03
	object	6.34 (0.58)			*sham*	7.12 (0.62)		

Significance of two-sided paired sample t-test: ^*^p < 0.05, ^***^p < 0.001. ^t^Statistical trend at 10% significance level. Mean (M), standard error (SE), significance (p) and 95%-confidence interval. *n* = 31.

**Figure 3:**
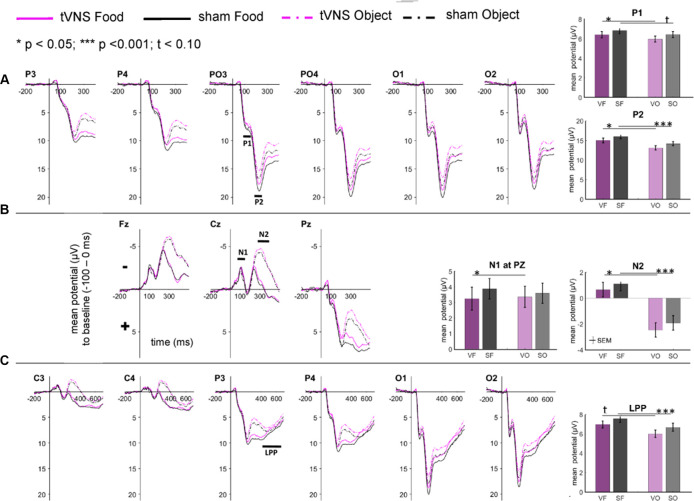
Effects of tVNS on ERPs calculated with frequential statistics. Channel **(A)** shows positive potentials (P1 and P2), channel **(B)** negative potentials (N1 and N2) and channel **(C)** LPP potential. Line plots: mean change in electrical potentials (μV) in relation to a baseline (−100 to stimulus onset) over the time (x-axis) are shown for the conditions (lines). Bar plots: mean potential for each task condition pooled across electrodes. Abbreviations describe task conditions (stimulation and picture type; VF = tVNS Food, SF = Sham Food, VO = tVNS Object, SO = Sham Object). Horizontal lines signify statistical main effects. ^t^*p* < 0.10, ^*^p < 0.05, ^***^p < 0.001.

**Figure 4:**
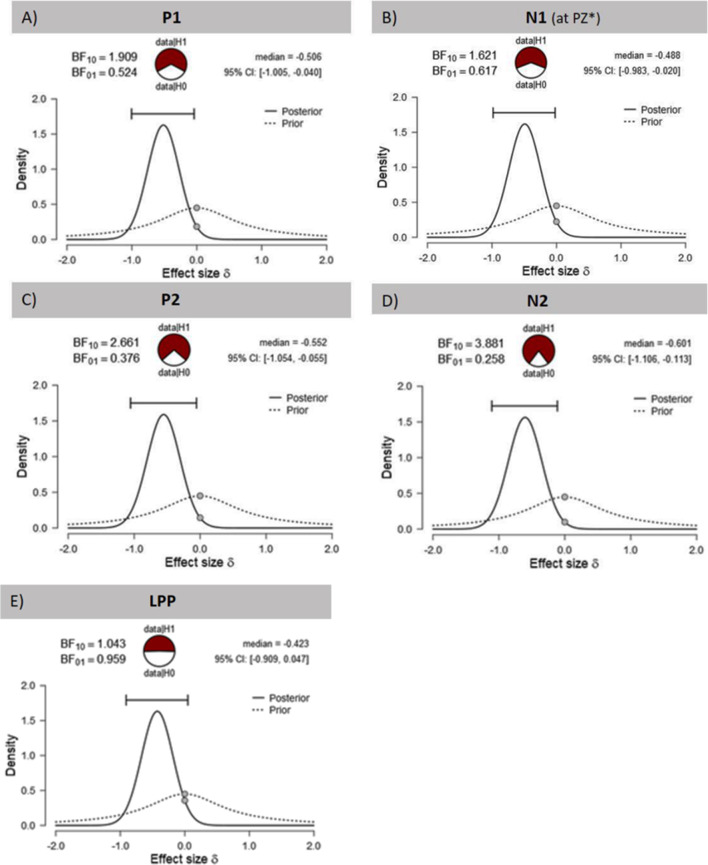
Effects of tVNS on ERPs calculated with Bayesian statistics. Graphics show the prior and posterior probability density for the stimulation main effect on **(A,B)** early latency phase components P1 and N1, **(C,D)** on middle latency phase components P2 and N2 and **(E)** on late latency phase component LPP. Cl describes the Bayesian credible interval. BF_(10)_ is the Bayes factor in favour to the alternative hypothesis (H1) and BF_(01)_ in favor of the null hypothesis. ^*^Result of exploratory analysis.

The visual inspection of the ERPs (Figure 3A) reveals the most pronounced effect for the P1 amplitudes at occipital electrode sites. The main effect stimulation reached significance (*F*_(1,30)_ = 5.36, *p* = 0.028, $\eta_{\text{par}}^{2}$ = 0.15) revealing lower amplitudes in the tVNS condition. The corresponding *BF*_(10)_ for this effect is 1.91 (Figure 4A). Food pictures evoked higher P1 amplitudes than object pictures did, although this difference reached only a statistical trend level (*F*_(1,30)_ = 3.98, *p* = 0.055, $\eta_{\text{par}}^{2}$ = 0.12). There was no significant interaction between the stimulation and picture factor (*F*_(1,30)_ = 0.12, *p* = 0.732, $\eta_{\text{par}}^{2}$ < 0.01) or any other factor.

Regarding the N1 component (Figure 3B), visual inspection suggested lower N1 amplitudes in response to food compared to object pictures and likewise higher amplitudes after tVNS compared to the control condition. Statistical analysis revealed, however, that the main effects for the factors picture and stimulation and their interaction were not significant. There was a trend effect for the interaction between the factors electrode × picture × stimulation (*F*_(2,29)_ = 3.22, *p* = 0.054, $\eta_{\text{par}}^{2}$ = 0.10). Exploratory *post hoc* ANOVA showed a significant stimulation main effect only at PZ electrode (*F*_(1,30)_ = 4.93, *p* = 0.034, $\eta_{\text{par}}^{2}$ = 0.14) with larger N1 amplitudes in the tVNS (*M* = 3.30, *SD* = 0.70) compared to the sham condition (*M* = 3.73, *SD* = 0.63). Inspected with Bayesian statistics, this main stimulation effect reached a *BF_(10)_* of 1.6 (Figure 4B).

Analyzing the later components P2 and N2 both ANOVAs revealed a significant stimulation main effect indicating smaller P2 amplitudes (Figure 3A) but higher N2 amplitudes (Figure 3B) for tVNS (P2: *F*_(1,30)_ = 6.21, *p* = 0.018, $\eta_{\text{par}}^{2}$ = 0.17; N2: *F*_(1,30)_ = 7.20, *p* = 0.012, $\eta_{\text{par}}^{2}$ = 0.19). *Post hoc* testing of these stimulation main effects showed small (P2, *BF*_(10)_ = 2.66) to moderate (N2, *BF*_(10)_ = 3.88) effect sizes (Figures 4C,D). Moreover, food pictures evoked higher P2 but smaller N2 amplitudes compared to object pictures (P2: *F*_(1,30)_ = 57.05, *p* < 0.001, $\eta_{\text{par}}^{2}$ = 0.66; N2: *F*_(1,30)_ = 384.32 *p* < 0.001, $\eta_{\text{par}}^{2}$ = 0.93). The interaction between the stimulation and any other factor was not significant for the P2, nor the N2-component (P2: *F*_(1,30)_ = 0.22, *p* = 0.643, $\eta_{\text{par}}^{2}$ = 0.01; N2: *F*_(1,30)_ = 0.22, *p* = 0.644, $\eta_{\text{par}}^{2}$ = 0.01).

Larger LPPs (Figure 3C) were observed in response to food compared to object pictures (*F*_(1,30)_ = 37.52 *p* ≤ 0.001, $\eta_{\text{par}}^{2}$ = 0.56). In the tVNS condition, LPP amplitudes were smaller than in the sham condition but the difference reached only a statistically trend level (*F*_(1,30)_ = 3.87 *p* = 0.059, $\eta_{\text{par}}^{2}$ = 0.11). The uncertainty is also reflected by a BF_(01)_ of 0.91 for the null and BF_(10)_ of 1.04 for the alternative hypothesis (see Figure 4E). No further significant interactions between the stimulation and any other factor were observed.

### Results on Food Consumption

The total food intake (in kcal); the consumption of protein, fat, and carbohydrates; and the duration of food consumption are given in Table 3. No stimulation effects were found, neither for the general food consumption nor the eaten amount of protein, fat, or carbohydrates.

**Table 3:** Descriptive food consumption statistics.

Food consumption	mdn (IQR)	mdn (IQR)	*t*_(30)_	*p*	g	BF_(10)_
	tVNS	Sham				
kCal (g)	1,307 (891–1,454)	1,144 (960–1,335)	0.10	0.92	0.02	0.19
Proteins (g)	39 (32–49)	39 (28–46)	0.20	0.49	0.26	0.20
Fat (g)	48 (36–59)	48 (41–59)	0.71	0.85	0.07	0.24
Carbohydrates (g)	144 (102–177)	138 (112–164)	0.94	0.36	0.39	0.29

*Medians (mdn), inter quartile ratio (IQR), test statistic (*t, p*) and effect size (Hedges’ g) and Bayes Factor (BF) for consumed kilocalories (kcal) and food ingredients for both conditions (tVNS, sham), *n* = 31*.

### Correlation of Electrophysiological Stimulation Effects With Food Consumption

**Figure 5:**
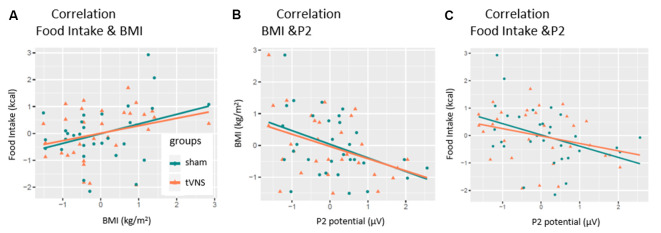
Correlation of P2-amplitude and behavioral outcomes. P2 amplitudes are pooled across electrodes and task conditions. Significant relations were found for **(A)** only sham condition. **(B)** Both conditions. **(C)** Only sham condition.

As expected, the number of consumed kilocalories correlated positively with the BMI in the control condition, but only as a statistical trend effect (*r* = 0.35, *p* = 0.057, see Figure 5A). Interestingly, this correlation was even weaker in the tVNS condition (*r* = 0.29, *p* = 0.112). As displayed in Figure 5B, P2 amplitudes (pooled across task conditions) and BMI correlated negatively in both stimulation conditions (tVNS: *r* = −0.40, *p* = 0.028; sham: *r* = −0.41, *p* = 0.021) while the amount of food intake (see Figure 5C and Supplementary Figure S1) correlated with the P2 amplitudes in the sham (*r* = −0.38 *p* = 0.035) but not in the tVNS condition (*r* = −0.291, *p* = 0.112). Corrected for multiple testing (0.05/5 = 0.01), all correlations missed statistical significance. However, the found relations are supported by the corresponding BFs (food intake and BMI: tVNS = 0.75, sham = 1.27; BMI and P2: tVNS = 2.27, sham = *2.85*; food intake and P2: tVNS = 0.7, sham = 2.27).

## Discussion

The present study used event-related potentials to test tVNS’ impact on food-related cognitive functions and whether these changes mediate food intake.

As hypothesized, our study shows that tVNS indeed has an impact on EEG components related to the processing of visual stimuli. TVNS significantly decreased P1 and P2 amplitudes and increased N2 amplitudes compared to sham stimulation. A similar trend was also seen for the LPP component. Against our assumptions, stimulation effects were food-stimulus-unspecific given that none of the expected interactions between stimulation condition and picture type reached significance. We also could not find any effects of tVNS on food intake. However, we did find a significant relationship between P2 amplitudes and the food consumption in sham but not with tVNS.

The reliability of our results is supported by the previously reported differential processing of food and object items (Plihal et al., 2001; Schacht et al., 2016; Carbine et al., 2018). The significant stimulation main effects are additionally confirmed by the results of Bayesian statistics. However, while large effect sizes were calculated using frequential statistics ($\eta_{\text{par}}^{2}$ range: 0.14–0.19), the results of Bayesian statistics indicated small to medium effect sizes (BF_(10)_ range: 1.6–3.88). It can therefore be assumed that the actual effect sizes are in the middle, since the results of Bayesian statistics can be regarded as the lower limit.

Stimulation effects on P1 and N1 suggest that tVNS already modulates initial sensory and visual attentional functions (Hillyard and Anllo-Vento, 1998; Bernat et al., 2001). More specifically, the reduced P1 and increased N1 potentials can be speculated to represent improved flexibility in attentional directing and/or allocation by reducing the costs of disengaging (P1) and an increased benefit in guiding attention (N1; Luck et al., 1990). This is corroborated by a study in which cervical VNS increased N1 amplitude in a working memory task in epilepsy patients (Sun et al., 2017).

P2 has been reported to be elevated in obese subjects (Nijs et al., 2010; Carbine et al., 2018), as well as in restraint-eaters in a hungry state (Plihal et al., 2001; Hachl et al., 2003), and to be decreased in the latter group after food intake. Thus, P2 was interpreted to indicate the arousing value of food (Sänger, 2019). Considering these findings, the decreased P2 amplitudes in the tVNS condition might indicate that tVNS reduces the processing of external stimuli unspecifically.

However, in our study, we also found a negative correlation between P2 amplitudes and food intake but only in the control group. This correlation is interesting because it implies a second factor by which the P2 component can be modulated—the blood glucose level. Previous research described reduced P2 amplitudes during hypoglycemic states in a food-unrelated cognitive task (Schultes et al., 2005; Svaldi et al., 2010). Presuming that the (preceding) blood glucose level influences the amount of food intake (during homeostatic eating), it is justified to infer (from our correlation) that the higher food intake after the examination indicates lower blood glucose levels (reflected by the P2 amplitudes) at the beginning of our examination. Importantly, that relation was not significant in the tVNS condition (see Figure 5), pointing to the discussed impact of tVNS on food metabolism and the modulation of satiety signaling (Banni et al., 2012; Malbert et al., 2017). This interpretation is in line with the increased P2 amplitudes observed in obese individuals since obesity is known to manifest higher blood glucose levels (Spiegelman et al., 1992). Taken together, our results imply that the P2 amplitudes are not only moderated by arousal—a fast switching reaction toward stimuli—but also by basic metabolic states. Unfortunately, we did not examine further somatic state variables as well as food intake-related behavior (hungriness, craving, wanting, liking; Berridge and Robinson, 1998) more closely.

The increased N2 amplitudes in the tVNS condition might tentatively suggest that tVNS enhances inhibitory processes and/or conflict monitoring (Carretié et al., 2004; Jonkman et al., 2007; Folstein and Van Petten, 2008) given prior findings on this component. This is in line with prior assumptions that (t)VNS modulates the NE-hormone release in the brain that is associated with neural inhibition (Henry, 2002; Fornai et al., 2011). Moreover, effects on conflict processing have already been reported by Fischer et al. (2018). This interpretation should be tested further in dedicated studies.

While visual inspection suggested a less pronounced LPP in the tVNS condition, this effect was neither robust with conventional statistics (*p* = 0.059) nor with Bayes statistics (*BF*_(01)_ = 1; *BF*_(10)_ = 1).

Finally, the fact that we did not find any effects on food intake can be explained by at least three reasons. First, in the present study normal-weight individuals were tested. However, Pardo et al. (2007) suggested that the VNS effects could be weight-dependent; the higher the initial body weight, the stronger the VNS effect on body weight. Second, we applied tVNS for approximately 2 h but studies showing effects on food intake (and body weight) stimulated much longer (weeks or months). Therefore, the time of stimulation could have been too short to reveal behavioral effects and we recommend long-term trials. Third, despite hunger, food intake is driven by a variety of reasons for example by habits or as a strategy for emotion regulation (Davidson et al., 2019). While habits should be constant in each participant, the personal stress level could have been varied. However, both could have overlaid possible effects on food intake—while the top-down regulated habits (“I always eat only a croissant in the morning”) overwrite bottom-up salience allocation and/or hunger feelings and therefore the assumed tVNS effects, the evaluation of the stress level before the test meal could have been a valuable covariate. Therefore, food intake behavior and the states of participants should be surveyed more precisely.

To summarize, while our study failed to reveal an effect of short-term tVNS on food consumption and differential processing of food pictures (i.e., no interaction between tVNS and picture type), a general effect on several ERP components was found that indicates a possible influence on attentional and inhibitory aspects in visual perception processes. We, therefore, suggest two lines of research for future studies. First, given the reported effects on weight in long-term invasive VNS, a longer-term intervention study seems to be justified. Second, the potential effects of tVNS on attentional and inhibitory cognitive functions need to be examined using dedicated paradigms. This information might also be important to judge potential side effects of this method of non-invasive brain stimulation.

## Data Availability Statement

The datasets generated for this study are available on request to the corresponding author.

## Ethics Statement

The studies involving human participants were reviewed and approved by the Ethics Committee of University of Lübeck. The patients/participants provided their written informed consent to participate in this study.

## Author Contributions

Experimental design was developed by MH, MT, and TM. Data were collected by MO and HA. Statistical analysis was performed by MO under supervision of MH and TM. Bayesian analysis was performed by MO. Manuscript was written by MO, MH, and TM. All authors read and approved the final manuscript.

## Conflict of Interest

The authors declare that the research was conducted in the absence of any commercial or financial relationships that could be construed as a potential conflict of interest.

## Footnotes

^1^https://fddb.info

^2^https://jasp-stats.org/

## References

[B1] Abubakr A. Wambacq I. (2008). Long-term outcome of vagus nerve stimulation therapy in patients with refractory epilepsy. J. Clin. Neurosci. 15, 127–129. 10.1016/j.jocn.2007.07.083 18068991

[B2] Afshin A. Forouzanfar M. H. Reitsma M. B. Sur P. Estep K. Lee A.. (2017). Health effects of overweight and obesity in 195 countries over 25 years. N. Engl. J. Med. 377, 13–27. 10.1056/NEJMoa1614362 28604169 PMC5477817

[B3] Al-Najim W. Docherty N. G. Le Roux C. W. (2018). Food intake and eating behavior after bariatric surgery. Physiol. Rev. 98, 1113–1141. 10.1152/physrev.00021.2017 29717927

[B4] Banni S. Carta G. Murru E. Cordeddu L. Giordano E. Marrosu F.. (2012). Vagus nerve stimulation reduces body weight and fat mass in rats. PLoS One 7:e44813. 10.1371/journal.pone.0044813 23028630 PMC3460935

[B5] Bell A. Sejnowski T. (1995). Information-maximization approach to blind separation and blind deconvolution. Neural Comput. 7, 1129–1159. 10.1162/neco.1995.7.6.1129 7584893

[B6] Bernat E. Bunce S. Shevrin H. (2001). Event-related brain potentials differentiate positive and negative mood adjectives during both supraliminal and subliminal visual processing. Int. J. Psychophysiol. 42, 11–34. 10.1016/s0167-8760(01)00133-7 11451477

[B7] Berridge K. C. Robinson T. E. (1998). What is the role of dopamine in reward: hedonics, learning, or incentive salience? Brain Res. Rev. 28, 308–367. 10.1016/s0165-0173(98)00019-8 9858756

[B8] Berthoud H. R. Neuhuber W. L. (2000). Functional and chemical anatomy of the afferent vagal system. Auton. Neurosci. 85, 1–17. 10.1016/S1566-0702(00)00215-0 11189015

[B9] Bodenlos J. S. Kose S. Borckardt J. J. Nahas Z. Shaw D. O’Neil P. M.. (2007). Vagus nerve stimulation acutely alters food craving in adults with depression. Appetite 48, 145–153. 10.1016/j.appet.2006.07.080 17081655

[B10] Bugajski A. J. Gil K. Ziomber A. Zurowski D. Zaraska W. Thor P. J. (2007). Effect of long-term vagal stimulation on food intake and body weight during diet induced obesity in rats. J. Physiol. Pharmacol. 58, 5–12. 17443024

[B11] Burneo J. G. Faught E. Knowlton R. Morawetz R. Kuzniecky R. (2002). Weight loss associated with vagus nerve stimulation. Neurology 59, 463–464. 10.1212/wnl.59.3.463 12177391

[B12] Butryn M. L. Webb V. Wadden T. A. (2011). Behavioral treatment of obesity. Psychiatr. Clin. North Am. 34, 841–859. 10.1016/j.psc.2011.08.006 22098808 PMC3233993

[B13] Carbine K. A. Christensen E. LeCheminant J. D. Bailey B. W. Tucker L. A. Larson M. J. (2017). Testing food-related inhibitory control to high- and low-calorie food stimuli: electrophysiological responses to high-calorie food stimuli predict calorie and carbohydrate intake. Psychophysiology 54, 982–997. 10.1111/psyp.12860 28338210

[B14] Carbine K. A. Rodeback R. Modersitzki E. Miner M. LeCheminant J. D. Larson M. J. (2018). The utility of event-related potentials (ERPs) in understanding food-related cognition: a systematic review and recommendations. Appetite 128, 58–78. 10.1016/j.appet.2018.05.135 29787830

[B15] Carretié L. Hinojosa J. A. Martín-Loeches M. Mercado F. Tapia M. (2004). Automatic attention to emotional stimuli: neural correlates. Hum. Brain Mapp. 22, 290–299. 10.1002/hbm.20037 15202107 PMC6871850

[B16] Chae J. H. Nahas Z. Lomarev M. Denslow S. Lorberbaum J. P. Bohning D. E.. (2003). A review of functional neuroimaging studies of vagus nerve stimulation (VNS). J. Psychiatr. Res. 37, 443–455. 10.1016/s0022-3956(03)00074-8 14563375

[B17] Colzato L. S. Ritter S. M. Steenbergen L. (2018). Transcutaneous vagus nerve stimulation (TVNS) enhances divergent thinking. Neuropsychol. 111, 72–76. 10.1016/j.neuropsychologia.2018.01.003 29326067

[B18] Davidson T. L. Jones S. Roy M. Stevenson R. J. (2019). The cognitive control of eating and body weight: it’s more than what you “think”. Front. Psychol. 10:62. 10.3389/fpsyg.2019.00062 30814963 PMC6381074

[B19] De Lartigue G. (2016). Role of the vagus nerve in the development and treatment of diet-induced obesity. J. Physiol. 594, 5791–5815. 10.1113/JP271538 26959077 PMC5063945

[B20] Delorme A. Makeig S. (2004). EEGLAB: an open source toolbox for analysis of single-trial EEG dynamics including independent component analysis. J. Neurosci. Methods 134, 9–21. 10.1016/j.jneumeth.2003.10.009 15102499

[B58] De Silva A. Salem V. Matthews P. M. Dhillo W. S. (2012). The use of functional MRI to study appetite control in the CNS. Exp. Diabetes Res. 2012:764017. 10.1155/2012/764017 22719753 PMC3376546

[B21] Ellrich J. (2011). Transcutaneous vagus nerve stimulation. Eur. Neurol. Rev. 6, 254–256. 10.17925/ENR.2011.06.04.254

[B22] Ferrario C. R. Labouèbe G. Liu S. Nieh E. H. Routh V. H. Xu S.. (2016). Homeostasis meets motivation in the battle to control food intake. J. Neurosci. 36, 11469–11481. 10.1523/JNEUROSCI.2338-16.2016 27911750 PMC5125214

[B23] Fischer R. Ventura-Bort C. Hamm A. Weymar M. (2018). Transcutaneous vagus nerve stimulation (tVNS) enhances conflict-triggered adjustment of cognitive control. Cogn. Affect. Behav. Neurosci. 18, 680–693. 10.3758/s13415-018-0596-2 29693214

[B24] Folstein J. R. Van Petten C. (2008). Influence of cognitive control and mismatch on the N2 component of the ERP: a review. Psychophysiology 45, 152–170. 10.1111/j.1469-8986.2007.00602.x 17850238 PMC2365910

[B25] Fornai F. Ruffoli R. Giorgi F. S. Paparelli A. (2011). The role of locus coeruleus in the antiepileptic activity induced by vagus nerve stimulation. Eur. J. Neurosci. 33, 2169–2178. 10.1111/j.1460-9568.2011.07707.x 21535457

[B26] Frangos E. Ellrich J. Komisaruk B. (2015). Non-invasive access to the vagus nerve central projections *via* electrical stimulation of the external ear: fMRI evidence in humans. Brain Stimul. 8, 624–636. 10.1016/j.brs.2014.11.018 25573069 PMC4458242

[B27] Gelman A. Hill J. Yajima M. (2012). Why we (usually) don’t have to worry about multiple comparisons. J. Res. Educ. Effect. 5, 189–211. 10.1080/19345747.2011.618213

[B28] Ghani S. Vilensky J. Turner B. Tubbs R. S. Loukas M. (2015). Meta-analysis of vagus nerve stimulation treatment for epilepsy: correlation between device setting parameters and acute response. Childs Nerv. Syst. 31, 2291–2304. 10.1007/s00381-015-2921-1 26493055

[B29] Gil K. Bugajski A. Thor P. (2011). Electrical vagus nerve stimulation decreases food consumption and weight gain in rats fed a high-fat diet. J. Physiol. Pharmacol. 62, 637–646. 22314566

[B30] Göbel C. H. Tronnier V. M. Münte T. F. (2017). Brain stimulation in obesity. Int. J. Obes. 41, 1721–1727. 10.1038/ijo.2017.150 28663570

[B31] Gorgulho A. A. Pereira J. L. B. Krahl S. Lemaire J. J. De Salles A. (2014). Neuromodulation for eating disorders. Obesity and anorexia. Neurosurg. Clin. N. Am. 25, 147–157. 10.1016/j.nec.2013.08.005 24262906

[B32] Hachl P. Hempel C. Pietrowsky R. (2003). ERPs to stimulus identification in persons with restrained eating behavior. Int. J. Psychophysiol. 49, 111–121. 10.1016/s0167-8760(03)00099-0 12919714

[B33] Han L. Jian-Bin Z. Chen X. Qing-Qing T. Wei-Xing S. Jing-Zhu Z.. (2015). Effects and mechanisms of auricular vagus nerve stimulation on high-fat-diet-induced obese rats. Nutrition 31, 1416–1422. 10.1016/j.nut.2015.05.007 26429664

[B34] Henry T. R. (2002). Therapeutic mechanisms of vagus nerve stimulation. Neurology 59, S3–S14. 10.1212/wnl.59.6_suppl_4.s3 12270962

[B35] Hillyard S. A. Anllo-Vento L. (1998). Event-related brain potentials in the study of visual selective attention. Proc. Natl. Acad. Sci. U S A 95, 781–787. 10.1073/pnas.95.3.781 9448241 PMC33798

[B36] Jacobs H. I. L. Riphagen J. M. Razat C. M. Wiese S. Sack A. T. (2015). Transcutaneous vagus nerve stimulation boosts associative memory in older individuals. Neurobiol. Aging 36, 1860–1867. 10.1016/j.neurobiolaging.2015.02.023 25805212

[B37] Johnson R. L. Wilson C. G. (2018). A review of vagus nerve stimulation as a therapeutic intervention. J. Inflamm. Res. 11, 203–213. 10.2147/jir.s163248 29844694 PMC5961632

[B38] Jongkees B. J. Immink M. A. Finisguerra A. Colzato L. S. (2018). Transcutaneous vagus nerve stimulation (tVNS) Enhances response selection during sequential action. Front. Psychol. 9:1159. 10.3389/fpsyg.2018.01159 30034357 PMC6043681

[B39] Jonkman L. M. Sniedt F. L. F. Kemner C. (2007). Source localization of the Nogo-N2: a developmental study. Clin. Neurophysiol. 118, 1069–1077. 10.1016/j.clinph.2007.01.017 17368096

[B73] Kass R. E. Raftery A. E. (1995). Bayes factors. J. Am. Stat. Asso. 90, 773–795. 10.1080/01621459.1995.10476572

[B40] Kakkar A. K. Dahiya N. (2015). Drug treatment of obesity: current status and future prospects. Eur. J. Intern. Med. 26, 89–94. 10.1016/j.ejim.2015.01.005 25634851

[B41] Kennedy J. E. (2015). Beware of inferential errors and low power with Bayesian analyses: power analysis is needed for confirmatory research. J. Parapsychol. 79, 53–64.

[B42] Kong F. Zhang Y. Chen H. (2015). Inhibition ability of food cues between successful and unsuccessful restrained eaters: a two-choice oddball task. PLoS One 10:e0120522. 10.1371/journal.pone.0133942 25886063 PMC4401785

[B43] Lomarev M. Denslow S. Nahas Z. Chae J. H. George M. S. Bohning D. E. (2002). Vagus nerve stimulation (VNS) synchronized BOLD fMRI suggests that VNS in depressed adults has frequency/dose dependent effects. J. Psychiatr. Res. 36, 219–227. 10.1016/s0022-3956(02)00013-4 12191626

[B44] Lopez-Calderon J. Luck S. J. (2014). ERPLAB: an open-source toolbox for the analysis of event-related potentials. Front. Hum. Neurosci. 8:213. 10.3389/fnhum.2014.00213 24782741 PMC3995046

[B45] Luck S. J. Heinze H. J. Mangun G. R. Hillyard S. A. (1990). Visual event-related potentials indexed focused attention within bilateral stimulus arrays. II. Functional dissociation of P1 and N1 components. Electroencephalogr. Clin. Neurophysiol. 75, 528–542. 10.1016/0013-4694(90)90139-b 1693897

[B72] Malbert C. H. Picq C. Divoux J. L. Henry C. Horowitz M. Love J. (2017). Obesity-Associated alterations in glucose metabolism are reversed by chronic bilateral stimulation of the abdominal vagus nerve. Diabetes 66, 848–857. 10.2337/db16-0847 28082456

[B46] Makeig S. Bell A. J. Jung T.-P. Sejnowski T. J. (1996). “Independent component analysis of electroencephalographic data,” in Advances in Neural Infomation Processing Systems (Vol. 8), eds Touretzky D. Mozer M. Hasselmo M. (Cambridge, MA: MIT Press), 145–151.

[B47] McClelland J. Bozhilova N. Campbell I. Schmidt U. (2013). A systematic review of the effects of neuromodulation on eating and body weight: evidence from human and animal studies. Eur. Eat. Disord. Rev. 21, 436–455. 10.1002/erv.2256 24155246

[B48] Nijs I. M. T. Franken I. H. A. Muris P. (2010). Food-related Stroop interference in obese and normal-weight individuals: behavioral and electrophysiological indices. Eat. Behav. 11, 258–265. 10.1016/j.eatbeh.2010.07.002 20850061

[B49] Pardo J. V. Sheikh S. A. Kuskowski M. A. Surerus-Johnson C. Hagen M. C. Lee J. T.. (2007). Weight loss during chronic, cervical vagus nerve stimulation in depressed patients with obesity: an observation. Int. J. Obes. 31, 1756–1759. 10.1038/sj.ijo.0803666 17563762 PMC2365729

[B50] Pelot N. A. Grill W. M. (2018). Effects of vagal neuromodulation on feeding behavior. Brain Res. 1693, 180–187. 10.1016/j.brainres.2018.02.003 29425906 PMC6003853

[B51] Plihal W. Haenschel C. Hachl P. Born J. Pietrowsky R. (2001). The effect of food deprivation on ERP during identification of tachistoscopically presented food-related words. J. Psychophysiol. 15, 163–172. 10.1027/0269-8803.15.3.163

[B52] Roslin M. Kurian M. (2001). The use of electrical stimulation of the vagus nerve to treat morbid obesity. Epilepsy Behav. 2, S11–S16. 10.1006/ebeh.2001.0213

[B53] Sainsbury A. Zhang L. (2012). Role of the hypothalamus in the neuroendocrine regulation of body weight and composition during energy deficit. Obes. Rev. 13, 234–257. 10.1111/j.1467-789x.2011.00948.x 22070225

[B54] Sänger J. (2019). Can’t take my eyes off you—How task irrelevant pictures of food influence attentional selection. Appetite 133, 313–323. 10.1016/j.appet.2018.11.030 30508612

[B55] Schacht A. Łuczak A. Pinkpank T. Vilgis T. Sommer W. (2016). The valence of food in pictures and on the plate: impacts on brain and body. Int. J. Gastr. Food Sci. 5–6, 33–40. 10.1016/j.ijgfs.2016.11.002

[B56] Schultes B. Kern W. Oltmanns K. Peters A. Gais S. Fehm H. L.. (2005). Differential adaptation of neurocognitive brain functions to recurrent hypoglycemia in healthy men. Psychoneuroendocrinology 30, 149–161. 10.1016/j.psyneuen.2004.06.007 15471613

[B57] Sellaro R. de Gelder B. Finisguerra A. Colzato L. S. (2018). Transcutaneous vagus nerve stimulation (tVNS) enhances recognition of emotions in faces but not bodies. Cortex 99, 213–223. 10.1016/j.cortex.2017.11.007 29275193

[B60] Sobocki J. Fourtanier G. Estany J. Otal P. (2006). Does vagal nerve stimulation affect body composition and metabolism? Experimental study of a new potential technique in bariatric surgery. Surgery 139, 209–216. 10.1016/j.surg.2005.06.025 16455330

[B59] Sobocki J. Thor P. Krolczyk G. Uson J. Diaz-Guemes I. Lipinski M. (2002). The cybergut. An experimental study on permanent microchip neuromodulation for control of gut function. Acta Chir. Belg. 102, 68–70. 10.1080/00015458.2002.11679268 12051092

[B61] Spiegelman D. Israel R. G. Bouchard C. Willett W. C. (1992). Absolute fat mass, percent body fat and body-fat distribution: which is the real determinant of blood pressure and serum glucose? Am. J. Clin. Nutr. 55, 1033–1044. 10.1093/ajcn/55.6.1033 1595574

[B62] Sun L. Peräkylä J. Holm K. Haapasalo J. Lehtimäki K. Ogawa K. H.. (2017). Vagus nerve stimulation improves working memory performance. J. Clin. Exp. Neuropsychol. 39, 954–964. 10.1080/13803395.2017.1285869 28492363

[B63] Svaldi J. Tuschen-Caffier B. Peyk P. Blechert J. (2010). Information processing of food pictures in binge eating disorder. Appetite 55, 685–694. 10.1016/j.appet.2010.10.002 20946926

[B64] Val-Laillet D. Aarts E. Weber B. Ferrari M. Quaresima V. Stoeckel L. E.. (2015). Neuroimaging and neuromodulation approaches to study eating behavior and prevent and treat eating disorders and obesity. Neuroimage Clin. 8, 1–31. 10.1016/j.nicl.2015.03.016 26110109 PMC4473270

[B65] Val-Laillet D. Biraben A. Randuineau G. Malbert C. H. (2010). Chronic vagus nerve stimulation decreased weight gain, food consumption and sweet craving in adult obese minipigs. Appetite 55, 245–252. 10.1016/j.appet.2010.06.008 20600417

[B66] Van Bockstaele E. J. Peoples J. Valentino R. J. (1999). Anatomic basis for differential regulation of the rostrolateral peri- locus coeruleus region by limbic afferents. Biol. Psychiatry 46, 1352–1363. 10.1016/s0006-3223(99)00213-9 10578450

[B67] Versace F. Kypriotakis G. Basen-Engquist K. Schembre S. M. (2016). Heterogeneity in brain reactivity to pleasant and food cues: evidence of sign-tracking in humans. Soc. Cogn. Affect. Neurosci. 11, 604–611. 10.1093/scan/nsv143 26609106 PMC4814789

[B68] Vijgen G. H. E. J. Bouvy N. D. Leenen L. Rijkers K. Cornips E. Majoie M.. (2013). Vagus nerve stimulation increases energy expenditure: relation to brown adipose tissue activity. PLoS One 8:e77221. 10.1371/journal.pone.0077221 24194874 PMC3806746

[B69] Wagenmakers E. J. Marsman M. Jamil T. Ly A. Verhagen J. Love J.. (2018). Bayesian inference for psychology. Part I: theoretical advantages and practical ramifications. Psychon. Bull. Rev. 25, 35–57. 10.3758/s13423-017-1343-3 28779455 PMC5862936

[B70] Wagenmakers E. J. Verhagen J. Ly A. (2016). How to quantify the evidence for the absence of a correlation. Behav. Res. Methods 48, 413–426. 10.3758/s13428-015-0593-0 26148822 PMC4891395

[B74] Warren C. M. Tona K. D. Ouwerkerk L. van Paridon J. Poletiek F. van Steenbergen H.. (2019). The neuromodulatory and hormonal effects of transcutaneous vagus nerve stimulation as evidenced by salivary alpha amylase, salivary cortisol, pupil diameter, and the P3 event-related potential. Brain Stimulation 12, 635–642. 10.1016/j.brs.2018.12.224 30591360

[B71] Watson T. D. Garvey K. T. (2013). Neurocognitive correlates of processing food-related stimuli in a Go/No-go paradigm. Appetite 71, 40–47. 10.1016/j.appet.2013.07.007 23892319

[B75] World Health Organization (WHO) (2020). Obesity and overweight. Available online at: https://www.who.int/en/news-room/fact-sheets/detail/obesity-and-overweight.

