# Supplementary material for: Effect of Short-Term Transcutaneous Vagus Nerve Stimulation (tVNS) on Brain Processing of Food Cues: An Electrophysiological Study
Source: Front Hum Neurosci. 2020 Jun 18;14:206. doi: 10.3389/fnhum.2020.00206 (PMC7314996; doi:10.3389/fnhum.2020.00206)
Supplement: Supplementary file 1 [file Data_Sheet_1.docx]

| **Table S1.** Breakfast Ingredients | | |
| --- | --- | --- |
| 2 multi seed rolls | |  |
| 2 rye bread rolls | |  |
| 1 white bread roll | |  |
| 1 croissant |  |  |
| 1 slice of white bread | |  |
| 3 slices of whole wheat bread | | |
| 5 portions á 25 g of margarine | | |
| 2 portions á 40g of curd (20% fat) | | |
| 1 portion á 20g of apricot jam | | |
| 3 slices of maasdamer cheese | | |
| 5 slices of butter cheese | |  |
| 2 portions of cream cheese natural | | |
| 3 portions of cream cheese with herbs | | |
| 2 apples |  |  |
| 1 banana |  |  |
| 1 strawberry milk à 500 ml | | |
