# Supplementary figures and images for: Effect of Short-Term Transcutaneous Vagus Nerve Stimulation (tVNS) on Brain Processing of Food Cues: An Electrophysiological Study
Source: Front Hum Neurosci. 2020 Jun 18;14:206. doi: 10.3389/fnhum.2020.00206 (PMC7314996; doi:10.3389/fnhum.2020.00206)

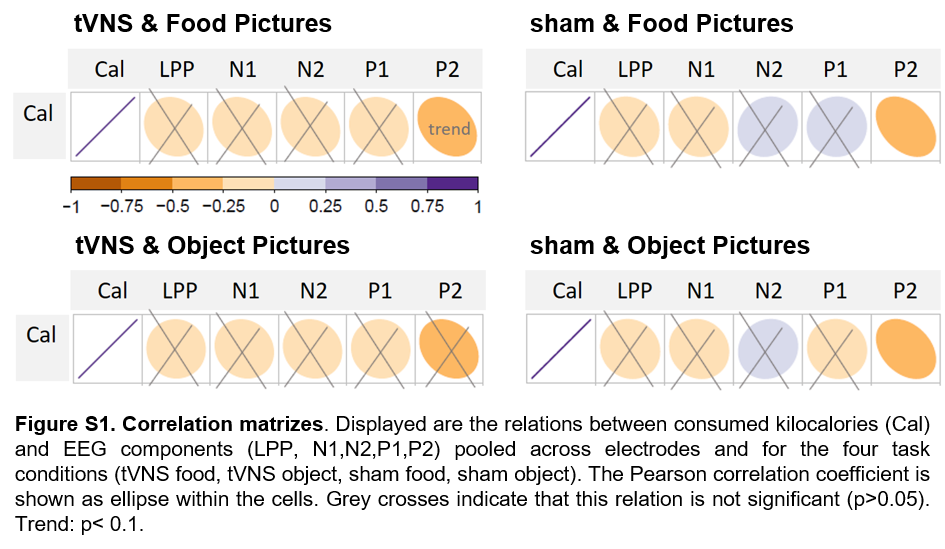

Supplement: Supplementary file 2 [file Image_1.tif]
